# Selective Loss of MATR3 in Spinal Interneurons, Upper Motor Neurons and Hippocampal CA1 Neurons in a MATR3 S85C Knock-In Mouse Model of Amyotrophic Lateral Sclerosis

**DOI:** 10.3390/biology11020298

**Published:** 2022-02-12

**Authors:** Justin You, Katarina Maksimovic, Jooyun Lee, Mashiat Khan, Rintaro Masuda, Jeehye Park

**Affiliations:** 1 Genetics and Genome Biology Program, The Hospital for Sick Children, Toronto, ON M5G 0A4, Canada; justin.you@mail.utoronto.ca (J.Y.); kat.maksimovic@mail.utoronto.ca (K.M.); jooyun.lee@mail.utoronto.ca (J.L.); mashiat.khan@mail.utoronto.ca (M.K.); r.masuda@mail.utoronto.ca (R.M.); 2 Department of Molecular Genetics, University of Toronto, Toronto, ON M5G 0A4, Canada

**Keywords:** amyotrophic lateral sclerosis (ALS), MATR3 S85C mutation, MATR3 S85C knock-in mice, neuropathology, motor neurons, spinal cord, spinal interneurons, hippocampus, cortex, reduced MATR3 immunoreactivity

## Abstract

**Simple Summary:**

Amyotrophic lateral sclerosis (ALS) is a neurodegenerative disease affecting the motor neurons in the brain and spinal cord. Mutations in the gene *Matr3* have been linked to ALS, including the autosomal dominant missense mutation S85C. We previously created a mouse model containing the S85C mutation in the *Matr3* gene to understand how it causes ALS. The S85C mice exhibited MATR3 staining loss in selective populations of degenerating neurons, such as Purkinje cells in the cerebellum and α-motor neurons in the lumbar spinal cord. However, studies have shown that neurons other than motor neurons may be involved in contributing to ALS; therefore, we investigated additional neuronal cell types in the spinal cord and brain. Here, we found that MATR3 staining is selectively reduced in interneurons and α-motor neurons of the cervical and thoracic regions of the spinal cord, as well as in subsets of upper motor neurons and hippocampal neurons. These neurons did not exhibit cell body loss; however, how the MATR3 loss affects neuronal function remains to be determined. Overall, these findings demonstrate that the MATR3 S85C mutation affects other neuronal types of the brain and spinal cord in addition to motor neurons, suggesting that these additional neuronal types are involved in ALS pathogenesis.

**Abstract:**

The neuropathological hallmark of amyotrophic lateral sclerosis (ALS) is motor neuron degeneration in the spinal cord and cortex. Accumulating studies report that other neurons in the central nervous system (CNS) are also affected in ALS. Mutations in *Matr3*, which encodes a nuclear matrix protein involved in RNA splicing, have been linked to ALS. Previously, we generated a MATR3 S85C knock-in (KI) mouse model that recapitulates early-stage features of ALS. We reported that MATR3 S85C KI mice exhibit defects in lumbar spinal cord motor neurons and in cerebellar Purkinje cells, which are associated with reduced MATR3 immunoreactivity. Here, we show that neurons in various other regions of the CNS are affected in MATR3 S85C KI mice. Using histological analyses, we found selective loss of MATR3 staining in α-motor neurons, but not γ-motor neurons in the cervical and thoracic spinal cord. Loss of MATR3 was also found in parvalbumin-positive interneurons in the cervical, thoracic and lumbar spinal cord. In addition, we found the loss of MATR3 in subsets of upper motor neurons and hippocampal CA1 neurons. Collectively, our findings suggest that these additional neuronal types may contribute to the disease process in MATR3 S85C KI mice.

## 1. Introduction

Amyotrophic lateral sclerosis (ALS) is a progressive neurodegenerative disease leading to muscle weakness and paralysis. Since ALS has been historically defined as a disease of lower motor neurons in the spinal cord and upper motor neurons in the motor cortex [1], most studies have focused on understanding the disease mechanism underlying the degeneration of these motor neurons [2]. However, accumulating studies indicate that other neurons in the CNS are also affected in ALS. Previous studies have found that spinal inhibitory interneurons that control motor neurons are affected in the post-mortem tissue of ALS patients and animal models [3,4,5]. In addition, neuropathology was reported in the cortex, hippocampus and cerebellum in ALS patients and animal models [2,6,7,8,9]. These studies highlight that non-motor neuronal cell types may contribute to the disease process. As ALS is increasingly being recognized as a multisystem neurodegenerative disease, studies investigating the contribution of cell types other than motor neurons to ALS pathogenesis are required. 

*MATR3* is a recently identified ALS gene, which encodes a nuclear matrix protein [10,11]. MATR3 has two zinc finger domains and two RNA recognition motifs, and has also been demonstrated to play a role in RNA splicing [12,13]. Several familial mutations in *Matr3* have been identified in ALS, including the autosomal dominant S85C missense mutation [11]. To understand how this mutation causes ALS, we previously generated a MATR3 S85C knock-in (KI) mouse model (*Matr3^S85C/S85C^*) that harbors the S85C mutation in the endogenous mouse *Matr3* gene [14]. The KI mice retain the regulatory regions of the *Matr3* gene and express the mutant form of MATR3 at the physiological level. These mutant mice recapitulate key features of early-stage ALS, including progressive motor deficits, muscle weakness, motor neuron defects and Purkinje cell loss [14]. *Matr3^S85C/S85C^* mice begin to exhibit motor deficits at 10 weeks of age (early disease stage), which progressively worsen at 30 weeks of age (advanced disease stage) and then reaching the disease end stage at 60 weeks (Supplementary Figure S5). There is no apparent difference in disease progression between males and females. Despite the ubiquitous expression of MATR3 throughout the CNS, we have found selective loss of MATR3 staining in degenerating neurons [14], suggesting that loss of MATR3 function contributes to neurodegeneration. Intriguingly, the loss of MATR3 was associated with Purkinje cell loss [14]. In addition, loss of MATR3 was observed only in α-motor neurons, but not γ-motor neurons in the lumbar spinal cord [14]. Among different motor neuron subtypes, α-motor neurons are large neurons that innervate extrafusal muscle fibers of skeletal muscle and are the most vulnerable in ALS patients, whereas γ-motor neurons are smaller neurons that innervate muscle spindle intrafusal fibers and are spared in ALS patients [15]. Since our findings in *Matr3^S85C/S85C^* mice showed that degenerating neurons are associated with a loss of MATR3, reduced MATR3 staining could be an indicator of S85C-affected neurons. Therefore, we sought to investigate whether other neuronal subtypes display a loss of MATR3 in *Matr3^S85C/S85C^* mice.

Here, we found several neuronal populations that exhibit loss of MATR3 in various CNS regions in *Matr3^S85C/S85C^* mice. The affected neurons include α-motor neurons in the ventral horn of the cervical and thoracic spinal cord, parvalbumin-positive (PVALB^+^) interneurons throughout the spinal cord, subsets of upper motor neurons in the cortex and hippocampal neurons in the CA1 region. However, the number of cell bodies of these neurons was not significantly affected. The loss of MATR3 suggests that these neurons may be involved in the disease process.

## 2. Materials and Methods

### 2.1. Animals

All mouse procedures were performed under the approval of the Animal Care Committee (ACC) at The Centre for Phenogenomics (TCP). *Matr3^S85C/S85C^* mice were generated as previously described [14]. We crossed heterozygous (*Matr3^S85C/+^*) mice to generate wildtype (*Matr3^+/+^*), heterozygous (*Matr3^S85C/+^*) and homozygous (*Matr3^S85C/S85C^*) littermates. This study only used the wildtype and homozygous littermates, which were aged to 10 weeks (early disease stage), 30 weeks (advanced disease stage), and 60 weeks (disease end stage) (Supplementary Figure S5). Unless otherwise specified, both male and female mice were used without discrimination.

### 2.2. Tissue Preparation

Mice were transcardially perfused using 4% paraformaldehyde (PFA, Sigma-Aldrich, St. Louis, MO, USA) in 1X phosphate-buffered saline (PBS, Roche, Mannheim, Germany). Brains and spinal columns were fixed for 48 h at room temperature in 4% PFA in PBS, then washed 3 times for 10 min in PBS. For cryoembedding, the spinal cord was dissected from spinal columns, cryoprotected in 30% sucrose in PBS for 48 h at 4 °C and embedded using the Optimal Cutting Temperature (OCT, Fisher HealthCare, Houston, TX, USA) compound. The cervical (C3–5), thoracic (T6–8), and lumbar regions (L3–5) were used for sectioning. Brains were embedded in paraffin as previously described [14,16]. 

### 2.3. Immunofluorescence on Cryosections

Sections from spinal cords embedded in OCT were washed in 0.3% Triton-X-100 (Sigma-Aldrich, St. Louis, MO, USA) in PBS (PBST), 3 times for 5 min. Sections were blocked in 5% normal donkey serum in PBST for 1 h at room temperature. Sections were incubated with primary antibody diluted in blocking buffer for 48 h at 4 °C. The following primary antibodies were used: MATR3 C-term (rabbit polyclonal, ab84422, 1:400, Abcam, Cambridge, UK), NeuN (mouse monoclonal, MAB377, 1:200, Millipore, Burlington, VT, USA), ChAT (goat polyclonal, NBP1-30052, 1:200, Novus Biologicals, Littleton, NL, USA), PVALB (goat, PVG-213, 1:1000, Swant, Burgdorf, Switzerland), GFAP (rabbit polyclonal, ab7260, 1:500, Abcam, Cambridge, UK), IBA1 (rabbit polyclonal, Wako, Osaka, Japan 019-19741, 1:1000). Sections were washed 3 times for 5 min with PBST and incubated with secondary antibodies diluted in blocking buffer for 3 h at room temperature (488/555/647 Alexa Fluor donkey anti-rabbit/mouse/goat IgG (H + L), 1:500 from ThermoFisher Scientific, Waltham, MA, USA). Sections were washed with PBST and then incubated with DAPI (D6210, 1:1000, Millipore, Burlington, VT, USA) for 15 min at room temperature. Sections were washed with PBST once and mounted with ProLong Gold Antifade mountant (ThermoFisher Scientific, Waltham, MA, USA P36930). All slides were imaged using Nikon A1R confocal microscope (Nikon Instruments, Melville, NY, USA).

### 2.4. Immunofluorescence on Paraffin Sections

Paraffin sections were rehydrated and washed for 5 min for each step using xylene, 100% ethanol, 95% ethanol, 70% ethanol, H_2_O, and PBS. Heat-induced epitope retrieval was performed with sodium citrate buffer (pH 6.0, in PBS). Sections were permeabilized using 0.05% Tween-20 (Sigma-Aldrich, St. Louis, MO, USA) in PBS (PBST), then blocked for 1 h at room temperature using 10% normal donkey serum and 0.25% Triton X-100 in PBST. Sections were incubated with primary antibody diluted in blocking buffer overnight at 4 °C. The following primary antibodies were used: MATR3 C-term (rabbit polyclonal, ab84422, 1:400, Abcam, Cambridge, UK), NeuN (mouse monoclonal, MAB377, 1:200, Millipore, Burlington, VT, USA), CTIP2 (rat monoclonal, ab18465, 1:250, Abcam, Cambridge, UK). Sections were washed with PBST 3 times, 5 min each, and incubated with secondary antibodies (488/555 Alexa Fluor donkey anti-rabbit/mouse IgG (H + L), 1:500 from ThermoFisher Scientific, Waltham, MA, USA) and DAPI (D6210, 1:1000, Millipore, Burlington, VT, USA) for 1 h at room temperature. Sections were washed with PBST 3 times, 5 min each, and mounted with ProLong Gold Antifade mountant (ThermoFisher Scientific, Waltham, MA, USA P36930). All slides were imaged using an SP8 Leica confocal microscope (Leica Microsystems, Wetzlar, Germany).

### 2.5. Imaging Spinal Motor Neurons and Interneurons and Quantification

The 40 µm transversely cryosectioned cervical and thoracic spinal cords were stained and imaged. The 20 µm transversely cryosectioned lumbar spinal cords were stained and imaged. For spinal motor neuron quantification, images of the ventral horn were acquired at 20× magnification. For interneuron quantification, images of the spinal cord grey matter were acquired using 20× magnification tiled scanning. Motor neurons were manually counted in the ventral horns based on ChAT and NeuN staining (α-motor neurons are ChAT^+^ NeuN^+^; γ-motor neurons are ChAT^+^ NeuN^−^). Interneurons were manually counted based on intense staining of PVALB. Neurons with visible nuclei were manually counted, and those with reduced MATR3 staining were manually counted based on staining levels relative to surrounding cells. Values from 3 sections were averaged per animal.

### 2.6. Hippocampal Neurons with Reduced MATR3 Staining Quantification

The 5 µm sagittal brain sections in paraffin were stained and imaged. Images were acquired at 5× magnification, 1.2× zoom, and 63× magnification for zoomed-in representative images of the CA1 and CA3. MATR3^+^ cells were counted within a 94.39 µm × 94.39 µm box from the 5× images. The number of cells per μm^2^ were extrapolated to the number of cells per 0.01 mm^2^. Values from 4 sections were averaged per animal. 

### 2.7. Cortical Neurons with Reduced MATR3 Staining Quantification

The 5 µm sagittal brain sections in paraffin were stained and imaged. Images were acquired at 5× magnification, 1.2× zoom, and 63× magnification for zoomed-in representative images. Cells were counted within a 1132.70 µm × 141.59 µm box and 1415.87 µm × 141.59 µm box from the 5× images, one at each end of the cortex (ventral and dorsal, respectively). The number of cells per μm^2^ was averaged between the two boxes and extrapolated to the number of cells per 0.1 mm^2^. NeuN^+^ cells with reduced MATR3 staining were manually counted based on staining levels relative to the surrounding cells. Values from 4 sections were averaged per animal.

### 2.8. Upper Motor Neuron Quantification

The 10 µm coronally sectioned brains in paraffin were stained and imaged. A confocal z-series was imaged for each section at 5×, 20×, and 40× magnification, step size of 0.5 μm. The 40× images were used as representative images. The 20× images were taken where there was the most abundant CTIP2^+^ staining. Cells were counted within a 284.09 µm × 284.09 μm box from the 20× images, and the number of cells per μm^2^ was extrapolated to the number of cells per 0.1 mm^2^. CTIP2^+^ cells with reduced MATR3 staining were manually counted based on staining levels relative to the surrounding cells. Values from 4 sections were averaged per animal.

### 2.9. Quantification of IBA1 and GFAP Staining

The 40 µm transversely cryosectioned spinal cords were stained and imaged. The integrated densities of IBA1 and GFAP staining were quantified after subtracting the background using the rolling ball radius of 10.0 pixels using ImageJ. This value was divided by the area of the spinal cord. Values from 3 sections were averaged per animal.

### 2.10. Statistical Analysis

Statistical significance was determined using unpaired, two-tailed Student’s *t*-test. All statistical tests were performed using GraphPad Prism 8.3.0 software package (San Diego, AK, USA), and data are displayed as mean ± SEM, with each datapoint representing an animal: * *p* < 0.05, ** *p* < 0.01, *** *p* < 0.001, **** *p* < 0.0001, ns = not significant. A minimum of 3 animals per condition were used for each experiment.

## 3. Results

### 3.1. Selective Loss of MATR3 in α-Motor Neurons and Interneurons in the Spinal Cord of Matr3^S85C/S85C^ Mice

**Figure 1:**
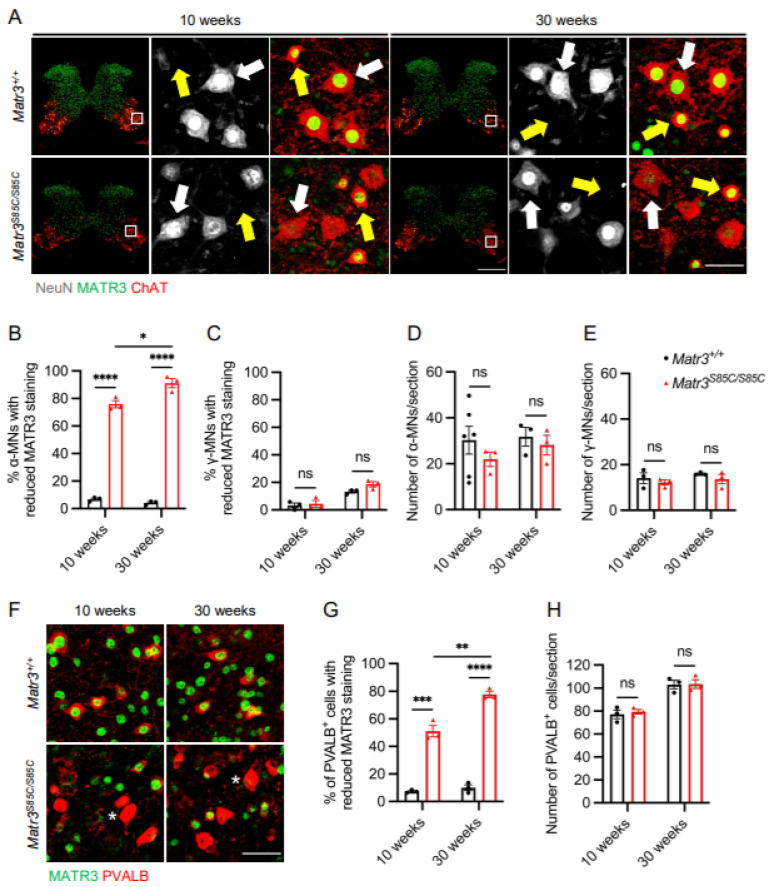
MATR3 loss in α-motor neurons and interneurons in the cervical spinal cord of *Matr3^S85C/S85C^* mice. (**A**) Representative images of 10 and 30 weeks cervical spinal cord staining for α-motor neurons (ChAT^+^, NeuN^+^, denoted by white arrows) and γ-motor neurons (ChAT^+^, NeuN^−^, denoted by yellow arrows). Scale bar for the spinal cord image denotes 500 μm and the zoomed-in image denotes 50 µm. Quantification of the percentage of motor neurons with reduced MATR3 staining in (**B**) α-motor neurons (10 weeks: *n* = 3 *Matr3^+/+^*, 3 *Matr3^S85C/S85C^*; 30 weeks: *n* = 3 *Matr3^+/+^*, 3 *Matr3^S85C/S85C^*) and (**C**) γ-motor neurons (10 weeks: *n* = 3 *Matr3^+/+^*, 3 *Matr3^S85C/S85C^*; 30 weeks: *n* = 3 *Matr3^+/+^*, 3 *Matr3^S85C/S85C^*). Quantification of the number of (**D**) α-motor neurons (10 weeks: *n* = 6 *Matr3^+/+^*, 3 *Matr3^S85C/S85C^*; 30 weeks: *n* = 3 *Matr3^+/+^*, 3 *Matr3^S85C/S85C^*) and (**E**) γ-motor neurons (10 weeks: *n* = 3 *Matr3^+/+^*, 3 *Matr3^S85C/S85C^*; 30 weeks: *n* = 3 *Matr3^+/+^*, 3 *Matr3^S85C/S85C^*). (**F**) Representative images of 10 and 30 weeks PVALB^+^ interneurons in the cervical spinal cord. Interneurons with reduced MATR3 staining are denoted by a white asterisk. Scale bar denotes 50 µm. (**G**) Quantification of the percentage of PVALB^+^ interneurons with reduced MATR3 staining (10 weeks: *n* = 3 *Matr3^+/+^*, 3 *Matr3^S85C/S85C^*; 30 weeks: *n* = 3 *Matr3^+/+^*, 3 *Matr3^S85C/S85C^*). (**H**) Quantification of the number of PVALB^+^ interneurons (10 weeks: *n* = 3 *Matr3^+/+^*, 3 *Matr3^S85C/S85C^*; 30 weeks: *n* = 3 *Matr3^+/+^*, 3 *Matr3^S85C/S85C^*). Bar graph heights depict mean ± SEM, with each datapoint representing an animal. * *p* < 0.05, ** *p* < 0.01, *** *p* < 0.001, **** *p* < 0.0001, ns = not significant.

**Figure 2:**
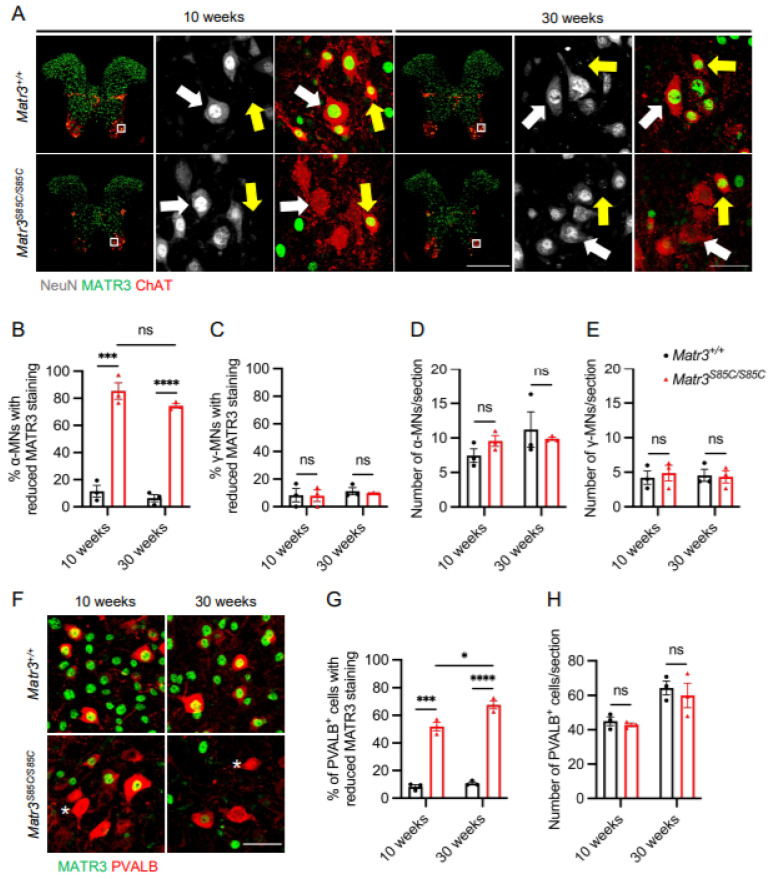
MATR3 loss in α-motor neurons and interneurons in the thoracic spinal cord of *Matr3^S85C/S85C^* mice. (**A**) Representative images of 10 and 30 weeks thoracic spinal cord staining for α-motor neurons (ChAT^+^, NeuN^+^, denoted by white arrows) and γ-motor neurons (Chat^+^, NeuN^−^, denoted by yellow arrows). Scale bar for the spinal cord image denotes 500 μm and the zoomed-in image denotes 50 µm. Quantification of the percentage of motor neurons with reduced MATR3 staining in (**B**) α-motor neurons (10 weeks: *n* = 3 *Matr3^+/+^*, 3 *Matr3^S85C/S85C^*; 30 weeks: *n* = 3 *Matr3^+/+^*, 3 *Matr3^S85C/S85C^*) and (**C**) γ-motor neurons (10 weeks: *n* = 3 *Matr3^+/+^*, 3 *Matr3^S85C/S85C^*; 30 weeks: *n* = 3 *Matr3^+/+^*, 3 *Matr3^S85C/S85C^*). Quantification of the number of (**D**) α-motor neurons (10 weeks: *n* = 3 *Matr3^+/+^*, 3 *Matr3^S85C/S85C^*; 30 weeks: *n* = 3 *Matr3^+/+^*, 3 *Matr3^S85C/S85C^*) and (**E**) γ-motor neurons (10 weeks: *n* = 3 *Matr3^+/+^*, 3 *Matr3^S85C/S85C^*; 30 weeks: *n* = 3 *Matr3^+/+^*, 3 *Matr3^S85C/S85C^*). (**F**) Representative images of 10 and 30 weeks PVALB^+^ interneurons in the thoracic spinal cord. Interneurons with reduced MATR3 staining are denoted by a white asterisk. Scale bar denotes 50 µm. (**G**) Quantification of the percentage of PVALB^+^ interneurons with reduced MATR3 staining (10 weeks: *n* = 3 *Matr3^+/+^*, 3 *Matr3^S85C/S85C^*; 30 weeks: *n* = 3 *Matr3^+/+^*, 3 *Matr3^S85C/S85C^*). (**H**) Quantification of the number of PVALB^+^ interneurons (10 weeks: *n* = 3 *Matr3^+/+^*, 3 *Matr3^S85C/S85C^*; 30 weeks: *n* = 3 *Matr3^+/+^*, 3 *Matr3^S85C/S85C^*). Bar graph heights depict mean ± SEM, with each datapoint representing an animal. * *p* < 0.05, *** *p* < 0.001, **** *p* < 0.0001, ns = not significant.

We have previously shown that MATR3 loss occurs in the α-motor neurons, but not γ-motor neurons in the lumbar spinal cord of *Matr3^S85C/S85C^* mice [14]. Therefore, we asked whether reduced MATR3 immunoreactivity is observed in α-motor neurons in the cervical and thoracic spinal cord (Supplementary Figure S1). We focused on two time points corresponding to the early disease stage (10 weeks) and advanced disease stage (30 weeks) (Supplementary Figure S5). Immunostaining results revealed significantly reduced MATR3 staining in α-motor neurons (ChAT^+^ NeuN^+^) but not in γ-motor neurons (ChAT^+^ NeuN^−^) in the cervical spinal cord of *Matr3^S85C/S85C^* mice at 10 and 30 weeks (Figure 1A–C). However, the number of cell bodies of α-motor neurons and γ-motor neurons in the cervical spinal cord was not significantly different between wildtype and *Matr3^S85C/S85C^* littermates (Figure 1D,E). Furthermore, these findings were consistent in the thoracic region of the spinal cord, in which selective loss of MATR3 was observed in α-motor neurons, but not γ-motor neurons (Figure 2A–C). In addition, we found no difference in the number of α-motor neurons and γ-motor neurons in the thoracic region (Figure 2D,E). These results are similar to our previous findings in the lumbar spinal cord [14]. Together, our findings indicate that α-motor neurons throughout the spinal cord are affected in the *Matr3^S85C/S85C^* mice.

Previous studies have shown that spinal interneurons are affected in ALS patients and mouse models [4,17,18,19,20]. Therefore, we investigated whether the spinal interneurons are also affected in *Matr3^S85C/S85C^* mice. First, we sought to examine whether reduced staining of MATR3 is present in the spinal interneurons of *Matr3^S85C/S85C^* mice. Using a general marker of interneurons, PVALB [21,22], we revealed a significant loss of MATR3 staining in PVALB^+^ interneurons in the cervical spinal cord of *Matr3^S85C/S85C^* mice at 10 and 30 weeks (Figure 1F,G). However, the number of interneurons in the cervical spinal cord of *Matr3^S85C/S85C^* mice was not significantly different from the wildtype mice (Figure 1H). Similarly, loss of MATR3 staining was also found in the interneurons of the thoracic spinal cord at 10 and 30 weeks, while the number of interneurons was not significantly different from that of the wildtype mice (Figure 2F–H). In addition, we found similar results in the lumbar spinal cord at 30 weeks (Supplementary Figure S2). These results indicate that the S85C mutation affects interneurons throughout the spinal cord. 

We previously reported the presence of neuroinflammation in the lumbar spinal cord and cerebellum of *Matr3^S85C/S85C^* mice [14]. Therefore, we conducted immunostaining to investigate whether microglial and astrocyte reactivity is increased in the cervical and thoracic spinal cord. Indeed, we found significantly increased levels of IBA1^+^ microglia and GFAP^+^ astrocytes in the cervical and thoracic spinal cord regions (Supplementary Figures S3 and S4), demonstrating that microglial and astrocytic reactivity is prevalent throughout the spinal cord. 

### 3.2. Selective Loss of MATR3 in the Subsets of Upper Motor Neurons in the Cortex of Matr3^S85C/S85C^ Mice

**Figure 3:**
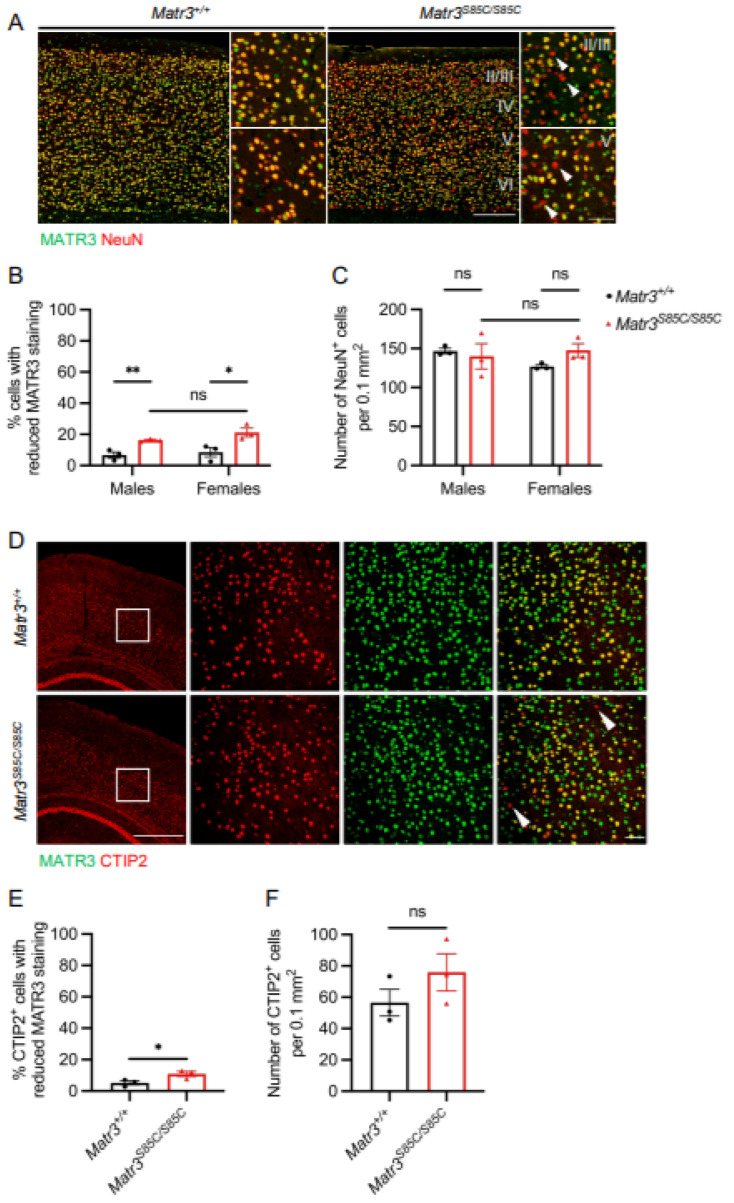
MATR3 loss in upper motor neurons and cortical neurons in *Matr3^S85C/S85C^* mice. (**A**) Representative images of the cortex with Roman numerals denoting the cortical layers. The scale bar for the full cortex images denotes 250 µm and the zoomed-in images denotes 50 µm. Arrowheads denote cells with reduced MATR3 staining. Brain sections are from 60-week-old mice. (**B**) Quantification of the number of NeuN^+^ cells with reduced MATR3 staining (males: *n* = 3 *Matr3^+/+^*, 3 *Matr3^S85C/S85C^*; females: *n* = 3 *Matr3^+/+^*, 3 *Matr3^S85C/S85C^*). (**C**) Quantification of the number of NeuN^+^ cells (males: *n* = 3 *Matr3^+/+^*, 3 *Matr3^S85C/S85C^*; females: *n* = 3 *Matr3^+/+^*, 3 *Matr3^S85C/S85C^*). (**D**) Representative images of the cortex stained with CTIP2 with scale bar denoting 500 µm (images on the left column). Representative enlarged images of the cortical layer V with the scale bar denoting 50 µm (images on the right three columns). Arrowheads denote cells with reduced MATR3 staining. Brain sections are from 30-week-old mice. (**E**) Quantification of the number of CTIP2^+^ cells with reduced MATR3 staining (*n* = 3 *Matr3^+/+^*, 3 *Matr3^S85C/S85C^*). (**F**) Quantification of the number of CTIP2^+^ cells (*n* = 3 *Matr3^+/+^*, 3 *Matr3^S85C/S85C^*). Bar graph heights depict mean ± SEM, with each datapoint representing an animal. * *p* < 0.05, ** *p* < 0.01, ns = not significant.

Given that the motor cortex is typically affected in ALS patients and mouse models [23,24], we investigated whether neurons in the brain cortex are affected in *Matr3^S85C/S85C^* mice using a NeuN antibody at disease end stage (60 weeks). Although there was no significant difference in the number of neurons in the cortex (Figure 3A,C), we found a small portion of neurons associated with reduced MATR3 staining in layers II/III and V in the cortex of the mutant mice (16.3% in males, 21.2% in females) (Figure 3A,B). To gain further insight into which specific neuronal types exhibit reduced MATR3 staining in layer V, where upper motor neurons reside, we conducted immunostaining at advanced disease stage (30 weeks) with MATR3 and CTIP2 antibodies, a marker of upper motor neurons [25]. We found a significant difference in the percentage of CTIP2^+^ cells with reduced MATR3 staining in the *Matr3^S85C/S85C^* mice (10.9% of cells) compared to the wildtype littermates (5.1% of cells) (Figure 3D,E); however, we did not find a significant loss of CTIP2^+^ cells in the mutant mice (Figure 3F). These results indicate that the S85C mutation affects a subset of upper motor neurons. However, the affected neuronal subtypes need to be identified and the functional consequences of MATR3 loss in these neurons need to be investigated.

### 3.3. Selective Loss of MATR3 in the Subsets of Hippocampal Neurons of Matr3^S85C/S85C^ Mice

**Figure 4:**
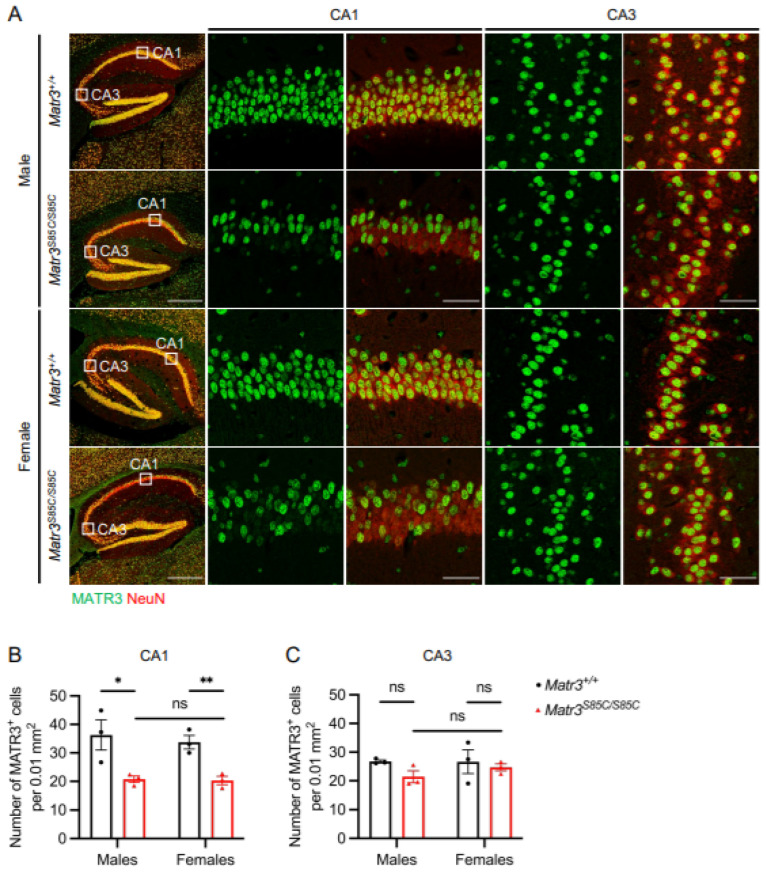
MATR3 loss in the hippocampal CA1 region of *Matr3^S85C/S85C^* mice. (**A**) Representative images of the entire hippocampus at 60 weeks with the scale bar denoting 500 µm (images on the left column). Representative enlarged images of the CA1 and CA3 with the scale bar denoting 50 µm (images on the right four columns). Quantification of the number of MATR3^+^ cells in the (**B**) CA1 (males: *n* = 3 *Matr3^+/+^*, 3 *Matr3^S85C/S85C^*; females: *n* = 3 *Matr3^+/+^*, 3 *Matr3^S85C/S85C^*) and (**C**) CA3 (males: *n* = 3 *Matr3^+/+^*, 3 *Matr3^S85C/S85C^*; females: *n* = 3 *Matr3^+/+^*, 3 *Matr3^S85C/S85C^*). Bar graph heights depict mean ± SEM, with each datapoint representing an animal. * *p* < 0.05, ** *p* < 0.01, ns = not significant.

We have previously reported that aged *Matr3^S85C/S85C^* male mice, but not females, show memory loss using the conditioned fear test [14]. To follow up on the neuropathological basis of these findings, we examined the hippocampus, the critical region linked to memory, to investigate MATR3 loss and neurodegeneration in *Matr3^S85C/S85C^* male and female mice at 60 weeks. We found that MATR3 staining was significantly reduced in subsets of neurons residing in the cornu ammonis field 1 (CA1) region (Figure 4A,B), but not in the CA3 region (Figure 4A,C), in both males and females. These results suggest that the MATR3 S85C mutation affects subsets of CA1 neurons in both males and females but may not explain the sex differences in memory loss. 

## 4. Discussion

Previously, we found that *Matr3^S85C/S85C^* mice show motor neuron defects and Purkinje cell loss associated with loss of MATR3 immunoreactivity [14], suggesting that loss of MATR3 function contributes to neurodegeneration. Here, we show loss of MATR3 in various CNS regions, including the cervical and thoracic spinal cord, cortex and hippocampus in *Matr3^S85C/S85C^* mice (Supplementary Figure S5), suggesting that additional neuronal populations are involved in the disease process of the mutant mice. Specifically, we found loss of MATR3 in α-motor neurons, but not in γ-motor neurons, in the cervical and thoracic spinal cord regions, consistent with what we previously found in the lumbar spinal cord region [14]. Furthermore, our results reveal that the number of α-motor neurons with MATR3 loss correlates with the severity of motor function as the percentage of α-motor neurons and interneurons with reduced MATR3 staining is higher at 30 weeks than at 10 weeks, with the exception of thoracic α-motor neurons. Together with our previous results [14], our findings demonstrate that α-motor neurons throughout the spinal cord are affected by the S85C mutation. Our findings support previous studies that demonstrate a selective vulnerability of α-motor neurons in post-mortem ALS tissues and animal models [15,26,27]. Although the majority of α-motor neurons showed loss of MATR3, there was no significant difference in the number of the cell bodies of α-motor neurons in the cervical and thoracic spinal cord between the mutant mice and the wildtype littermates, which is similar to what we observed in the lumbar region [14]. Furthermore, due to our previous findings of NMJ denervation, presynaptic axon blebbing and decreased endplate size in the tibialis anterior muscles of *Matr3^S85C/S85C^* mice [14], future studies will be required to determine whether NMJ defects are found in other skeletal muscle groups that are innervated by cervical and thoracic motor neurons. 

Spinal inhibitory interneurons are important for controlling lower motor neurons and are affected in ALS [3,4,5,28]. Studies on post-mortem ALS spinal cord tissue have reported the loss of PVALB^+^ interneurons [19]. Recent studies using *SOD1^G93A^* mouse models of ALS have shown the degeneration of inhibitory interneurons, and have demonstrated that dampening the spinal inhibitory neuron activity in the wildtype mice recapitulates the motor phenotype in *SOD1^G93A^* mice [4,17,18], supporting that inhibitory interneurons contribute to ALS pathogenesis. Our findings reveal that a subpopulation of PVALB^+^ interneurons exhibit loss of MATR3 throughout the spinal cord in *Matr3^S85C/S85C^* mice, suggesting that these interneurons are involved in the disease process in this model as well. However, we did not find significant degeneration of interneurons in the mutant mice. Additional experiments at the disease end stage (60 weeks) would be necessary to investigate whether the loss of MATR3 contributes to the degeneration of interneurons. Furthermore, future studies are needed to identify which subtypes of spinal interneurons are affected [22] and how their synaptic inputs onto motor neurons are impaired. Further investigation will allow us to better understand neuropathology in the spinal cord and its relationship to motor defects in ALS.

We have previously described increased microglial and astrocyte reactivity in the cerebellum and lumbar spinal cord of *Matr3^S85C/S85C^* mice. RNA-sequencing data also revealed that these regions are associated with the upregulation of immune-related genes at early disease stage (10 weeks of age) [14]. Here, we also found increased microglial and astrocyte reactivity within the cervical and thoracic spinal cord of *Matr3^S85C/S85C^* mice at 10 weeks of age. This finding likely suggests that neuroinflammation occurs early in the disease process throughout the spinal cord and correlates with the regions exhibiting loss of MATR3.

Upper motor neurons reside in layer V of the motor cortex and modulate and integrate connections between the cortex and spinal cord to provide cognitive input into motor functions [29]. Upper motor neuron pathology is found in post-mortem brain tissue of ALS patients [1,30]. In addition, several ALS mouse models, including *SOD1^G93A^* mice, show upper motor neuron pathology [24,31,32,33]. We report a significant but moderate reduction (10.9%) in MATR3 staining in the upper motor neurons in *Matr3^S85C/S85C^* mice. This moderate reduction is reflected in our previous study showing the lack of differentially expressed genes in the cortex in the early disease stage [14], suggesting that pathology in the cortex is likely not a substantial contributor to ALS phenotypes in *Matr3^S85C/S85C^* mice. However, identifying the subpopulation of upper motor neurons that are affected and whether the loss of MATR3 contributes to functional defects in these upper motor neurons requires further investigation. 

Previous studies have shown that ALS is also associated with a loss of hippocampal volume and hippocampal pathology [34,35,36]. Our previous study showed that *Matr3^S85C/S85C^* mice show male-specific memory loss in the conditioned fear test [14], which led us to examine MATR3 staining in the hippocampus of these mice. We found loss of MATR3 in subsets of neurons in the CA1 region of the hippocampus in both male and female *Matr3^S85C/S85C^* mice, with no difference between the sexes. Additionally, no loss of MATR3 was found in the CA3 region. These results reveal that selective loss of MATR3 occurs in the CA1 region in *Matr3^S85C/S85C^* mice. However, the implications of MATR3 loss in the CA1 region have yet to be determined. Considering the similar findings in male and female *Matr3^S85C/S85C^* mice, the memory impairment found specifically in males will need to be elucidated by investigating other regions involved in memory, such as the amygdala and entorhinal cortex.

## 5. Conclusions

Our findings reveal that MATR3 is selectively lost in α-motor neurons and interneurons throughout the spinal cord and in subpopulations of upper motor neurons and hippocampal CA1 neurons in the brain. These neuronal subtypes do not exhibit a significant loss in the number of neurons; however, how the loss of MATR3 affects neuronal function has yet to be determined. These findings are important for understanding the effect of the S85C mutation on these various neuronal types in the central nervous system and will guide future studies on how the S85C mutation causes ALS.

## Data Availability

The data presented in this study are available upon request from the corresponding author.

## Supplementary Materials

The following supporting information can be downloaded at: https://www.mdpi.com/article/10.3390/biology11020298/s1, Figure S1: Graphical representation of the murine spinal cord, Figure S2: MATR3 loss in the subsets of interneurons of *Matr3^S85C/S85C^* lumbar spinal cord, Figure S3: Increased microglial reactivity in *Matr3^S85C/S85C^* cervical and thoracic spinal cord, Figure S4: Increased astrocyte reactivity in *Matr3^S85C/S85C^* cervical and thoracic spinal cord, Figure S5: Timeline of disease progression in *Matr3^S85C/S85C^* mice.
