# Supplementary material for: Selective Loss of MATR3 in Spinal Interneurons, Upper Motor Neurons and Hippocampal CA1 Neurons in a MATR3 S85C Knock-In Mouse Model of Amyotrophic Lateral Sclerosis
Source: Biology (Basel). 2022 Feb 12;11(2):298. doi: 10.3390/biology11020298 (PMC8869279; doi:10.3390/biology11020298)
Supplement: Supplementary file 1 [file biology-11-00298-s001.zip › biology-1541855- Supplementary Figures.pdf]

Supplementary Figure S1

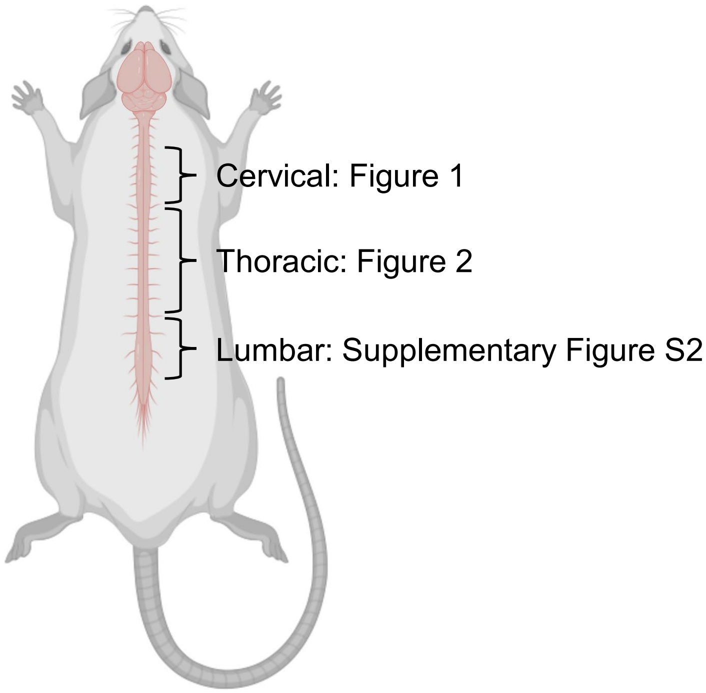

Supplementary Figure S1. Graphical representation of the murine spinal cord. The cervical, thoracic and lumbar spinal cord regions and reference to the appropriate figures are indicated.

Supplementary Figure S2

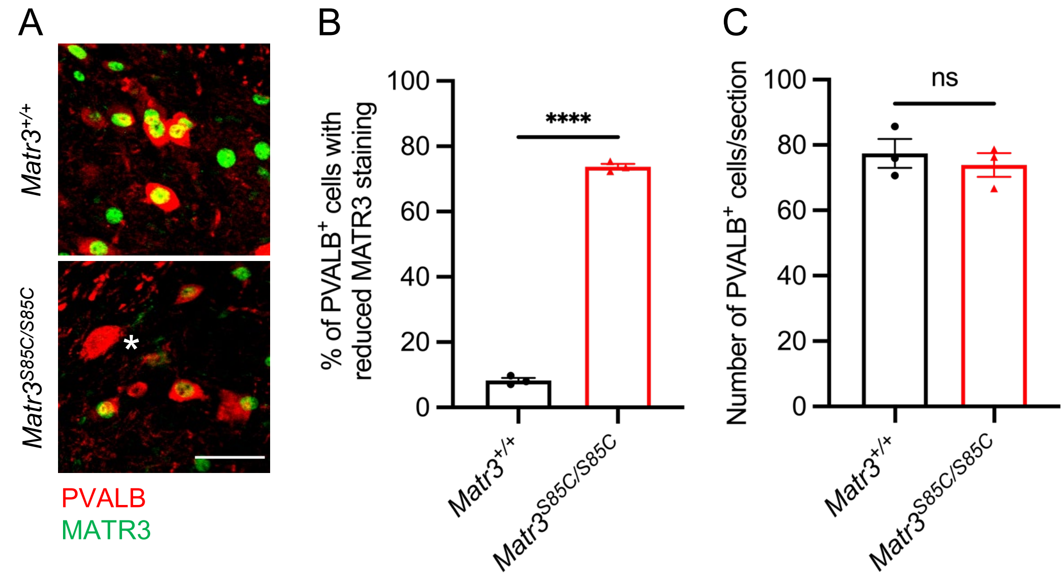

Supplementary Figure S2. MATR3 loss in the subsets of interneurons of *Matr3*$^{S85C/S85C}$ lumbar spinal cord. (A) Representative images of PVALB^{+} interneurons at 30 weeks. Interneurons with reduced MATR3 staining are denoted by a white asterisk. Scale bar denotes 50 μm. (B) Quantification of the percentage of PVALB^{+} interneurons with reduced MATR3 staining (n = 3 *Matr3*$^{+/+}$, 3 *Matr3*$^{S85C/S85C}$). (C) Quantification of the number of PVALB^{+} interneurons (n = 3 *Matr3*$^{+/+}$, 3 *Matr3*$^{S85C/S85C}$). Bar graph heights depict mean ± SEM, with each datapoint representing an animal. *$p < 0.05$, **$p < 0.01$, ***$p < 0.001$, ****$p < 0.0001$, ns = not significant.

Supplementary Figure S3

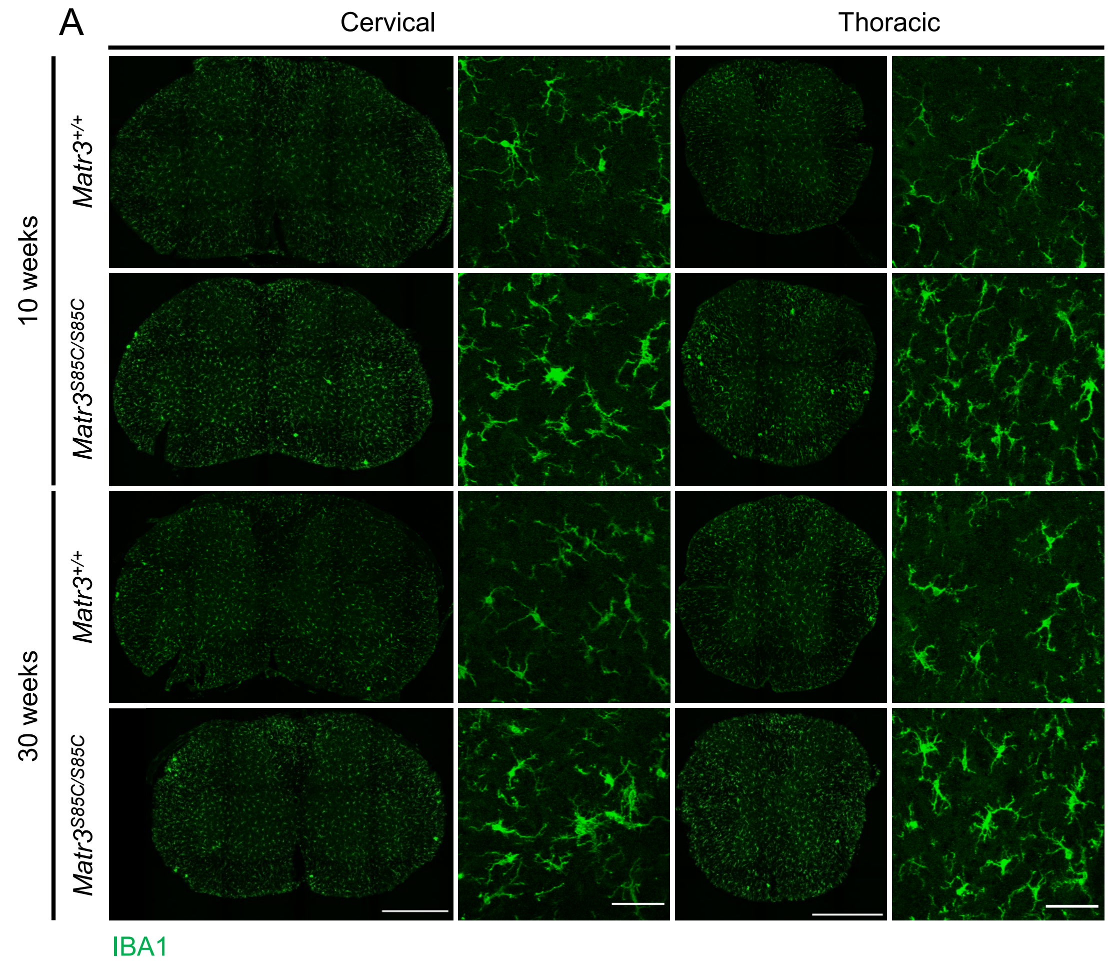

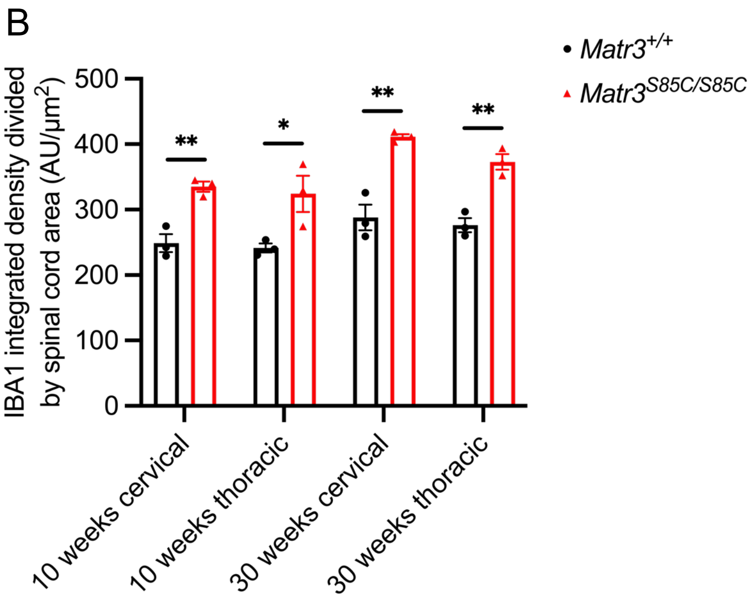

Supplementary Figure S3. Increased microglial reactivity in *Matr3*$^{S85C/S85C}$ cervical and thoracic spinal cord. (A) Representative images of microglial staining marked by IBA1. Scale bar of full spinal cord sections denotes 500 µm; scale bar of zoomed-in images of microglia in the ventral horn denotes 50 µm. (B) Quantification of IBA1 integrated density (10 weeks cervical: n = 3 *Matr3*$^{+/+}$, 3 *Matr3*$^{S85C/S85C}$; 10 weeks thoracic: n = 3 *Matr3*$^{+/+}$, 3 *Matr3*$^{S85C/S85C}$; 30 weeks cervical: n = 3 *Matr3*$^{+/+}$, 3 *Matr3*$^{S85C/S85C}$; 30 weeks thoracic: n = 3 *Matr3*$^{+/+}$, 3 *Matr3*$^{S85C/S85C}$). Bar graph heights depict mean ± SEM, with each datapoint representing an animal. *$p < 0.05$, **$p < 0.01$, ***$p < 0.001$, ****$p < 0.0001$, ns = not significant.

Supplementary Figure S4

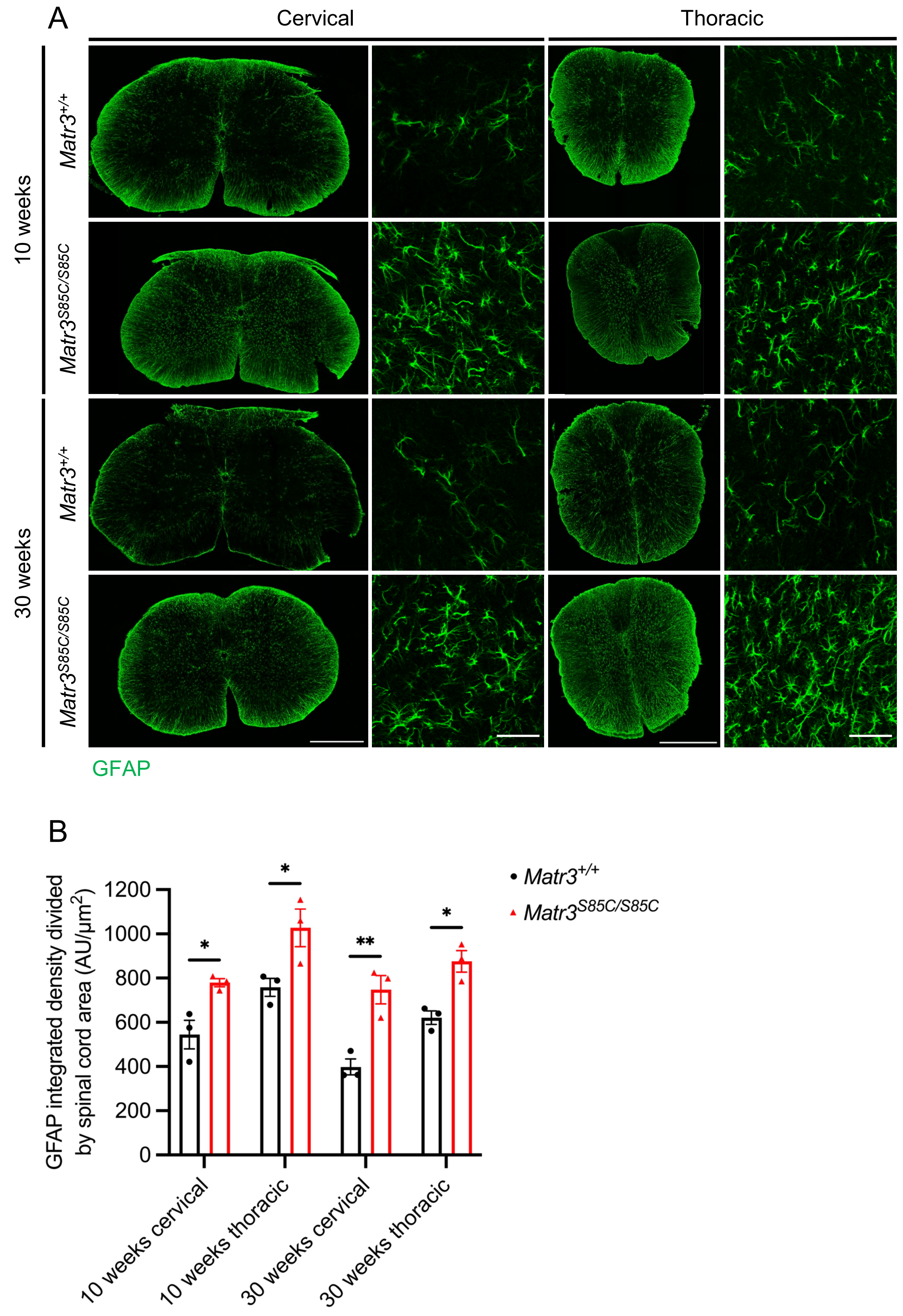

Supplementary Figure S4. Increased astrocyte reactivity in *Matr3*$^{S85C/S85C}$ cervical and thoracic spinal cord. (A) Representative images of astrocyte staining marked by GFAP. Scale bar of full spinal cord sections denotes 500 μm; scale bar of zoomed-in images of astrocytes in the ventral horn denotes 50 μm. (B) Quantification of GFAP integrated density (10 weeks cervical: n = 3 *Matr3*$^{+/+}$, 3 *Matr3*$^{S85C/S85C}$; 10 weeks thoracic: n = 3 *Matr3*$^{+/+}$, 3 *Matr3*$^{S85C/S85C}$; 30 weeks cervical: n = 3 *Matr3*$^{+/+}$, 3 *Matr3*$^{S85C/S85C}$; 30 weeks thoracic: n = 3 *Matr3*$^{+/+}$, 3 *Matr3*$^{S85C/S85C}$). Bar graph heights depict mean ± SEM, with each datapoint representing an animal. *$p < 0.05$, **$p < 0.01$, ***$p < 0.001$, ****$p < 0.0001$, ns = not significant.

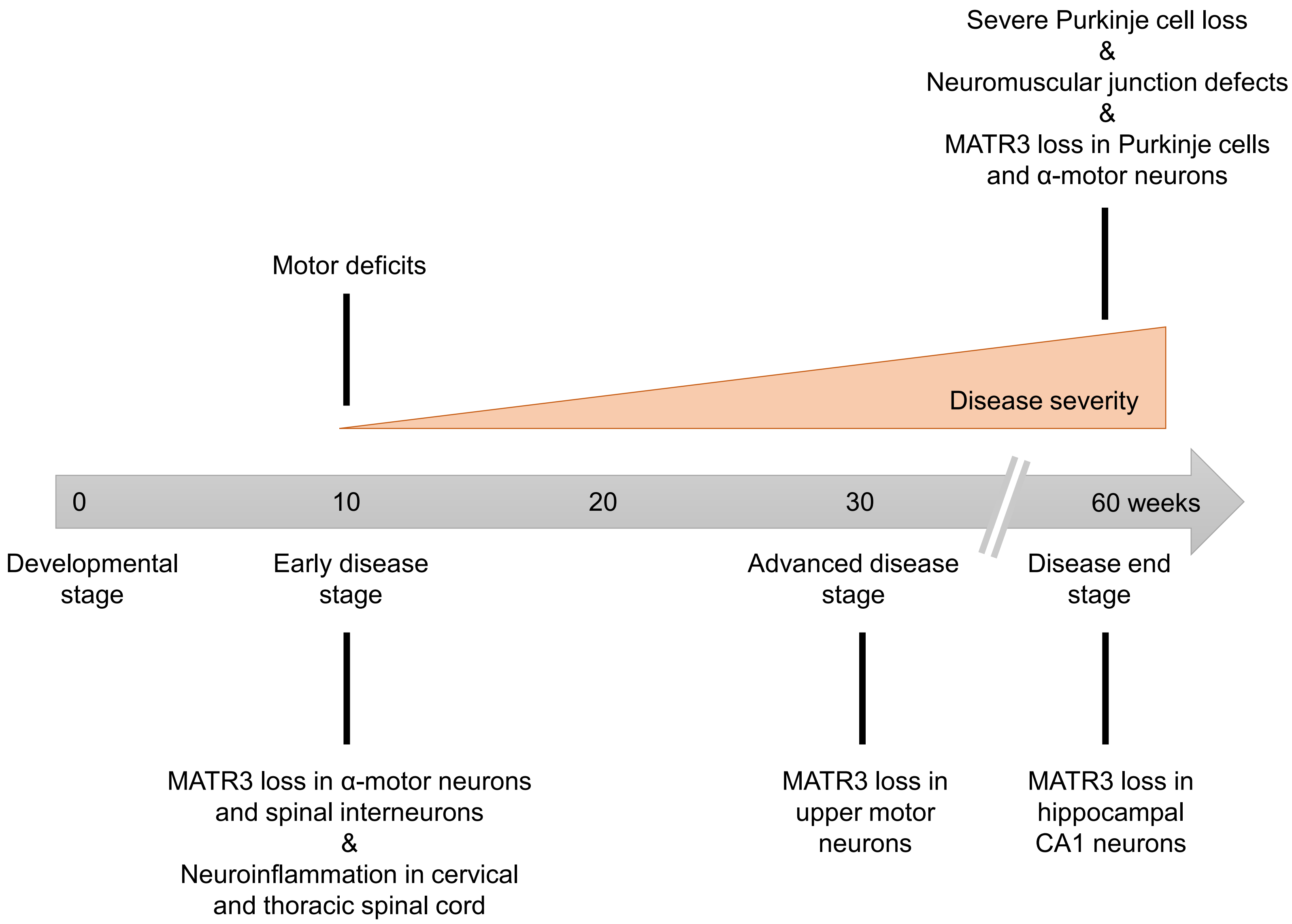

Supplementary Figure S5. Timeline of disease progression in *Matr3*$^{S85C/S85C}$ mice. Findings that were previously identified in Kao *et al*. *Nature Communications* 2020 are shown on the top portion of the graphic. Novel findings presented in this paper are shown on the bottom portion of the graphic.
